# Perceived quality of life in chronic obstructive pulmonary disease patients: a cross-sectional study in primary care on the role of illness perceptions

**DOI:** 10.1186/1471-2296-15-140

**Published:** 2014-08-03

**Authors:** Saskia WM Weldam, Jan-Willem J Lammers, Monique JWM Heijmans, Marieke J Schuurmans

**Affiliations:** 1 Department of Respiratory Diseases, Division Heart & Lungs, University Medical Center Utrecht, Utrecht, The Netherlands; 2 Department of Respiratory Diseases, Division Heart & Lungs, University Medical Center Utrecht, Utrecht, The Netherlands; 3 NIVEL Netherlands Institute for Health Services Research, NPCG: National Panel of the chronically ill and disabled, Utrecht, The Netherlands; 4 Department of Rehabilitation, Nursing Science & Sports, University Medical Center Utrecht, Utrecht, The Netherlands

**Keywords:** Chronic lung diseases, COPD, Psychological factors, Illness beliefs, Common Sense Model, Participation in daily life, Health-related quality of life, Primary care

## Abstract

**Background:**

Previous research has shown that in Chronic Obstructive Pulmonary Disease (COPD) patients, it is important to consider not only physical functioning and complaints but also psychological factors, such as illness perceptions, to explain differences in Health-Related Quality of Life (HRQoL). The objective of this study is to analyse the extent to which the specific dimensions of illness perceptions according to the Common Sense Model (corrected for airflow limitation, dyspnoea and comorbidities) contribute to HRQoL.

**Method:**

In a cross-sectional study in primary care, 90 COPD patients completed questionnaires: The Brief Illness Perception Questionnaire, the Medical Research Council dyspnoea scale, the Clinical COPD Questionnaire (CCQ) and the Chronic Respiratory Questionnaire (CRQ). Analyses were performed with multiple linear regression.

**Results:**

When corrected for confounders (airflow limitation, dyspnoea and comorbidities), *identity* (β = .42) and *comprehensibility* (β = -.16) were associated with HRQoL (CCQ). *Identity, comprehensibility* and dyspnoea explained 56% of the variation in HRQoL (R^2^ = .56). *Consequences* (β = -.50) and *treatment control* (β = .20) were associated with HRQoL (the CRQ’s physical domain). They explained 59% of the variation in the CRQ physical (R^2^ = .59) domain. *Treatment control* (β = .19) and e*motional response* (β = -.33) were associated with the CRQ emotional domain.

**Conclusions:**

Patients who experience fewer symptoms attributed to COPD, who have a better understanding of the disease, who experience less impact of COPD in daily life*,* who experience better treatment control and who have less of an emotional response have better HRQoL. This study indicates that the HRQoL of COPD patients is associated with illness perceptions as well as with the severity of dyspnoea as experienced by patients. Airflow limitation measures or comorbidities do not add to the explanation of HRQoL. The results of this study provide starting points for the development of interventions focusing on illness perceptions to support COPD patients in their disease management and to improve HRQoL.

## Background

Chronic Obstructive Pulmonary Disease (COPD) is a chronic disease characterised by progressive and persistent airflow limitation [1]. In the Netherlands, more than 360,000 people have been diagnosed with COPD [2]. The prevalence is estimated at 2.3% of men and 2.1% of women [2]. COPD patients may face limitations in daily activities and reduced quality of life caused by dyspnoea, airflow limitation, skeletal muscle dysfunction, and comorbidities [1,3]. Three major goals of COPD care and treatment are to reduce symptoms, increase participation in daily activities and improve health-related quality of life (HRQoL) [1]. In primary care settings in the Netherlands and many other countries, care for patients with COPD has increasingly moved from hospitals to primary care settings. Practice nurses have become essential in supporting COPD patients in their disease management [4].

**Figure 1:**
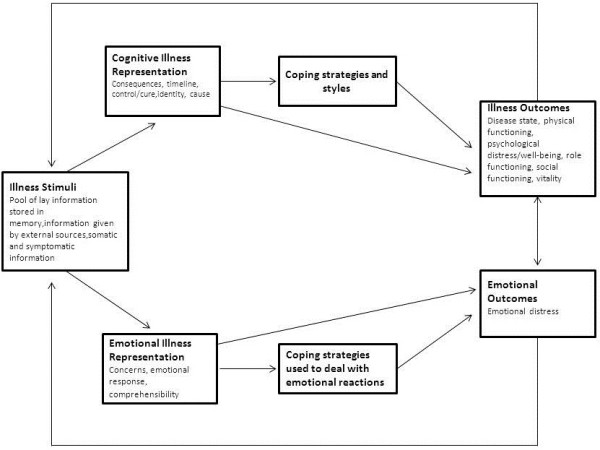
**The Common sense model (Adapted from Hagger et al. and Kaptein et al.) **[13,15]**.**

The pulmonary and extrapulmonary effects of the disease have an impact on physical, emotional, and mental well-being in COPD patients [5,6]. Although the assessment of COPD relies mainly on the degree of airflow limitation (i.e., the decrease in forced expiratory volume in one second (FEV1)), there is evidence that FEV1 has a relatively poor correlation with symptoms, HRQoL and daily functioning [7-9]. Therefore, other models in addition to strict medical models are increasingly used to explain differences in daily functioning and HRQoL in chronically ill patients. These models presume that biological factors as well as psychological and social factors play a significant role in the explanation of functioning and HRQoL in chronic illnesses [10,11]. One of the psychological factors that is considered important in this context is illness perceptions. Illness perceptions are the central concept of the Common Sense Model (CSM) [12,13]. This model suggests that people have personal beliefs about their illness that often do not match medical views but that nevertheless determine, to a large extent, how people respond to their illness. These illness perceptions include beliefs about consequences, the timeline of the disease, ability to control the disease and the extent to which the treatment helps in controlling the disease. They also include perceptions of symptoms attributed to the disease (identity), understanding of the disease, concerns and emotional response to the disease [12-14]. The CSM presumes that these various dimensions of illness perceptions are logically related to health behaviours and HRQoL. Therefore, these perceptions are considered key elements for understanding the ways that people attempt to manage threats to their health [12,13]. The CSM is depicted in Figure 1.

Previous research in outpatient and clinical populations has demonstrated that COPD patients who believe that the impact of the disease on daily life is less serious, who have positive beliefs about the treatment and who have less strong emotional reactions have better HRQoL than patients who have more negative beliefs [16-18]. Our previous study of COPD patients in a primary care setting, which explored the extent to which the combination of illness perceptions, proactive coping and depressive symptoms contribute to daily activities and HRQoL, revealed that illness perceptions are associated with HRQoL. More positive perceptions of illness were related to better HRQoL [9]. In this study, we will expand on our earlier research by analysing the extent to which the specific dimensions of illness perceptions contribute to HRQoL. Investigating these dimensions of illness perceptions in patients with mild to severe COPD allows us to test the assumption of the CS Model that specific beliefs about consequences, the timeline of the disease, ability to control the disease, ability to control the disease by treatment, symptoms, comprehensibility of the disease, emotional response and concerns are related to HRQoL.

## Method

### Study design and participants

This cross-sectional study was conducted in ten general practices throughout the Netherlands between June 2010 and April 2011. The study sample consisted of COPD patients who attended the participating general practices and visited the practice nurses during consulting- hours. The patients included in the study complied with the following criteria: a diagnosis of mild COPD (GOLD I), moderate COPD (GOLD II) or severe COPD (GOLD III) [1]. The GOLD (Global initiative for chronic Obstructive Lung Disease) is a classification of severity of airflow limitation in COPD based on post-bronchodilator forced expiratory volume in one second (FEV_1_) and the forced vital capacity (FVC) [1]. Furthermore, they had to be both physically and mentally able to complete the questionnaires. Patients were excluded if they had participated in another study or if they had a primary diagnosis of asthma. The Medical Research Ethics Committee (MREC) of the University Hospital Utrecht concluded that the Medical Research Involving Human Subjects Act (WMO) does not apply to this study; therefore, no WMO approval by the MREC was needed. The MREC ensured that the individuals involved in the study were adequately informed that their data would be used for research proposes. All participants provided written informed consent to participate in the study.

### Procedure

Eleven practice nurses working in ten participating general practices asked eligible COPD patients who visited them during consulting-hours if they would be willing to participate in the study. Eligible patients who expressed willingness to participate received a standardised letter explaining the aims of the study, particularly that the study would investigate the impact of COPD in daily life. After giving written informed consent, the participating patients completed the questionnaires at home and then returned them in a stamped addressed envelope to our centre.

### Measures

#### Illness perceptions

To assess the various dimensions of illness perceptions, the Brief Illness Perception Questionnaire (B-IPQ) [19] was used. This brief version was used because it is more suitable, less taxing, much quicker and much easier to complete than the long version IPQ-R [20]. The B-IPQ is a self-administered scale consisting of eight items on an 11-point scale (range 0–10). Each item represents a dimension of the CSM (Figure 1). Five items assess cognitive representations of the illness, and three items assess the emotional representation of the illness. The dimensions and implications of the scores are depicted in Table 1. A higher score on these dimensions implies that patients believed in a stronger influence of illness upon daily life *(“consequences”),* held stronger belief in a chronic time course *(“timeline”*), had greater perceived personal control of illness *(“personal control”),* had greater perceived control of the disease by treatment *(“treatment control”),* and had a greater experience of severe symptoms as a result of the illness *(“identity”)*. Two of the items assess emotional representations of illness. A higher score implies that patients had greater feelings of concern about the illness *(“concern”)* and a stronger emotional response to the illness *(“emotional response”)*. One questionnaire item assesses *“comprehensibility”*; a higher score implies a better understanding of the illness. One open-ended item assesses causal beliefs about COPD. This item asks patients to list their views on the three most important causal factors of their illness.

**Table 1:** **The dimensions of the Brief-Illness Perception Questionnaire (B-IPQ)**[19]

**Dimensions B-IPQ**	**Higher score implies:**
**Consequences**	Greater perceived influences of COPD
**Timeline**	A stronger belief in a chronic time course
**Personal control**	Greater perceived personal control
**Treatment control**	Greater perceived control by treatment
**Identity**	Greater experience of severe symptoms as a result of COPD
**Concern**	Greater feelings of concern about COPD
**Comprehensibility**	A better understanding of the illness

#### Dyspnoea

The MRC dyspnoea scale is a questionnaire that consists of six statements about perceived breathlessness: grade 0, “I don’t suffer from shortness of breath” grade 1, “I only get breathless with strenuous exercise”; grade 2, “I get short of breath when hurrying on the level or up a slight hill”; grade 3, “I walk slower than people of the same age on the level because of breathlessness or have to stop for breath when walking at my own pace on the level”; grade 4, “I stop for breath after walking 100 yards or after a few minutes on the level”; and grade 5, “ I am too breathless to leave the house” [21]. Patients selected the grade that applied to them.

#### Health-related quality of life

To measure HRQoL, the Clinical COPD Questionnaire (CCQ) [22] and the Chronic Respiratory Disease Questionnaire Self-Administered Short version (CRQ-SAS) [23] were used. The CCQ is a self-administered questionnaire consisting of 10 questions. Response options range from 0 (“no limitations/asymptomatic”) to 6 (“totally limited/extremely symptomatic”). The total score is computed by summing the scores and dividing the total score by the number of items. Higher scores indicate lower HRQoL.

The CRQ-SAS is a 20-item self-administered questionnaire covering four dimensions: dyspnoea, fatigue, emotional function and mastery. The response options for each question range from 1 (maximum impairment) to 7 (no impairment). The total score per domain is computed by summing the scores and dividing the total score by the number of items. In the analyses, the CRQ-SAS was divided into two domains: the CRQ-SAS physical domain (the mean of the dyspnoea and fatigue domains) and the CRQ-SAS emotional domain (the mean of the emotional function and mastery domains). Higher scores indicate better HRQoL.

In addition, data on pulmonary function were collected: FEV1 in litres and FVC in litres. To calculate the predicted forced expiratory volume percentage (FEV1% predicted), data on height and weight (according to the local general practitioner registry) were collected. Co-morbidities were measured by the Charlson comorbidity index [24]. This index is a validated method of classifying comorbidity from medical records and measures 17 conditions. The index score is the total of assigned weights and represents a measure of the burden of comorbidity [24,25].

Sociodemographic variables, such as age, gender, education level (based on the International Standard Classification of Education: ISCED) [26], working status, marital status and disease-related variables (medication use, smoking status) were also collected.

### Analyses

Descriptive statistics were used to present patients’ backgrounds, medical characteristics and their causal beliefs about COPD.

Linear regression analyses (adjusted for the confounders age, gender, dyspnoea, airflow reduction and comorbidities) were performed to quantify the associations between illness perceptions and HRQoL [27].

First, a crude model with the eight specific dimensions of illness perceptions (model 1) was analysed. In the second model (model 2), illness perceptions were corrected for the confounders of age and gender. In the third model (model 3), illness perceptions were adjusted for dyspnoea, airflow reduction (FEV1%predicted) and comorbidities. In the regression models, the standardised βs were used to compare the strength of the various independent variables. The adjusted explained variance (adjusted R2) per model was then analysed. Because only 3% of the data was missing, a complete case analysis was performed. All analyses were performed with the Statistical Package for the Social Sciences (SPSS 20.0 for Windows).

## Results

### Sample characteristics

A total of 98 patients completed the questionnaires. In the analyses, it was found that eight patients had no COPD; they had a GOLD stage of 0 with an FEV1/FVC ratio >70%. Therefore, these patients were excluded from the analyses. The sample characteristics of the 90 patients are presented in Table 2. The mean age of the patients in the study sample was 65, with a standard deviation (SD) of 9.0. Forty-six percent of study sample was women. Most patients (n = 60, 67%) had moderate COPD (GOLD grade II). Half of the patients were retired (51%). In the non-retired population (n = 44), 20 patients (22% from the total population) had full-time jobs. Most of the patients had a medium educational level (62%).

**Table 2:** Patient characteristics

**Variables**	**Total population (N = 90)**	
**Gender**		
Male	49 (54.4%)	
Female	41 (45.6%)	
**Age, years**		
Mean	65.19 (SD 9.0)	
≤ 50	5 (5.6%)	
51-60	26 (28.9%)	
61-70	31 (34.4%)	
71-80	23 (25.6%)	
> 80	5 (5.6%)	
**Years diagnosed with COPD Mean disease severity**		8.1 (SD 8.11)
GOLD^a^ I	18 (20.0%)	
GOLD II	60 (66.7%)	
GOLD III	12 (13.3%)	
FEV1 mean	1.9 (1.0-3.7) (SD .59)	
FEV1% predicted	67.0 (36.5-101.2) (SD 14.4)	
**Educational level**^**b**^		
Low	13 (14.4%)	
Medium	56 (62.3%)	
High	21 (23.3%)	
**Retired**	46 (51.1%)	
**Paid work (among ≤ 64 years)**	20 (22.2%)	
**Marital status**		
Married	59 (65.6%)	
Widowed	8 (8.9%)	
Divorced	8 (8.9%)	
Single	15 (16.7%)	
**Smoking status**		
Current smoker	36 (40.0%)	
Former smoker	49 (54.4%)	
Never smoked	5 (5.6%)	
**Medication use**	82 (91.1%)	
**Charlson comorbidity index, ≥1**	28 (31.1%)	

^a^Global Initiative for Chronic Obstructive Lung Disease.

^b^Categories are based on the International Standard Classification of Education (ISCED) [26].

### Illness perceptions and disease-related characteristics

The means, standard deviations, range of various dimensions of illness perceptions, dyspnoea, airflow limitations (FEV1), and HRQoL are presented in Table 3. In general, given their mean scores on the dimensions of illness perceptions, participants in this study considered their COPD chronic but not very serious, easily controlled by medical care or self-care, and with only minor consequences for daily life (Table 3). Most patients believe that smoking caused their COPD (71%), followed by heredity (22%), air pollution (16%) and allergy (8%).

**Table 3:** Descriptions of illness perceptions (B-IPQ), MRC dyspnoea, FEV1 and Health-Related Quality of Life (CCQ and CRQ) (N = 88–90)

	**Mean (SD)**	***Range***	***Ref range***	***Percentage***
Illness perceptions (B-IPQ)				
Consequences	3.6 (2.5)	0-9	0–10	
Timeline	9.1 (2.1)	0-10	0–10	
Personal control	6.0 (2.5)	0-10	0–10	
Treatment control	6.8 (2.7)	0-10	0–10	
Identity	4.0 (2.6)	0-10	0–10	
Concern	4.1 (3.1)	0-10	0–10	
Comprehensibility	7.6 (2.7)	0-10	0–10	
Emotional response	3.0 (3.1)	0-10	0–10	
*Causes of COPD*				
Smoking				71%
Heredity				22%
Air pollution				16%
Allergy				8%
Other				42%
MRC dyspnoea	1.7 (1.0)	0-5	0-6	
FEV1 (litres)	1.9 (0.6)	1.0-3.7		
FEV% pred	67.0 (14.4)	36.5-101.2		
HRQoL: CCQ	1.4 (0.8)	0.0-3.8	0-6	
HRQoL: CRQ:				
CRQ-SAS physical	5.6 (1.3)	2.70-7.00	1-7	
CRQ-SAS emotional	5.60 (1.0)	2.73-7.00	1-7	

B-IPQ = Brief Illness Perception Questionnaire, CCQ- = Clinical COPD Questionnaire, CRQ-SAS = Chronic Respiratory Disease Questionnaire Self-Administered Short version, CRQ-SAS physical = CRQ-SAS dyspnoea domain and CRQ-SAS fatigue domain, CRQ-SAS emotional = CRQ-SAS emotional domain and CRQ-SAS mastery domain, FEV1 = Forced Expiratory Volume in 1 second, FEV%pred = Forced Expiratory Volume Percentage from predicted, HRQoL = Health-Related Quality of Life, MRC dyspnoea = Medical Research Council dyspnoea scale.

### Illness perceptions and HRQoL

As shown in the crude regression model, which is not corrected for confounders (model 1, Table 4), *consequences* (β = .26), *identity* (β = .43) and *comprehensibility* (β = -.16) were associated with HRQoL as measured by the CCQ. When corrected for the confounders of age and gender (model 2), only *identity* (β = .44) was associated with HRQoL. In model 3, corrected for the confounders of dyspnoea, FEV1%predicted and comorbidities, *identity* (β = .42) and *comprehensibility* (β = -.16) were associated with HRQoL. *Identity, comprehensibility* and dyspnoea explained 56% of the variation in HRQoL in model 3 (R^2^ = .56). FEV%pred and comorbidity were not associated with the CCQ. These results indicate that COPD patients with weaker perceptions of *identity* and greater understanding *(comprehensibility)* of the disease have better HRQoL.

**Table 4:** Regression models between various illness perception items and dependent variable health-related quality of life (CCQ) N = 86

	**Model 1 (Block 1)**		**Model 2 (Block 1 and 2)**		**Model 3 (Block 1 and 3)**	
	**R**^**2**^	**β**	**R**^**2**^	**β**	**R**^**2**^	**β**
**Block 1: Perceptions**	.53		.54		.56	
Consequences		.26^*^		.26		.21
Timeline		.01		–.01		–.03
Personal control		.03		.02		.04
Treatment control		–.11		–.16		–.15
Identity		.43^**^		.44^**^		.42^**^
Illness concern		.02		.03		–.01
Comprehensibility		–.16^*^		–.13		–.16*
Emotional response		.16		.09		.13
**Block 2: Demographic characteristics**						
Age				.06		
Gender				.10		
**Block 3: Clinical characteristics**						
MRC dyspnoea						.23^**^
FEV%pred						.01
Comorbidity						–.10
**F change model**		13.9^***^		1.00		2.3^***^

R^2^ is an adjusted R^2^. β is a standardised β.

*P ≤ 0.05; ** P ≤ 0.01; *** p < 0.001.

FEV%pred = forced expiratory volume percentage from predicted, MRC dyspnoea = Medical Research Council dyspnoea scale.

As shown in Table 5 (model 1 and model 2), *consequences* (β = -.55) and *treatment control* (β = .16) were associated with HRQoL as measured by the CRQ-SAS physical domain. When corrected for the confounders of dyspnoea, FEV1%predicted and comorbidity (Table 5, model 3), *consequences* (β = -.50) and *treatment control* (β = .20) were associated with the CRQ-SAS’s physical domain. *Consequences, treatment control* and dyspnoea explained 59% of the variation in the CRQ-SAS’s physical domain (R^2^ = .59). These results indicate that COPD patients with weaker perceived consequences and more perceived effectiveness of the treatment have better HRQoL as measured by the CRQ-SAS’s physical domain (the mean of the dyspnoea and fatigue domains).

**Table 5:** Regression models between various illness perceptions items and dependent variable health-related quality of life (CRQ physical) N = 87

	**Model 1 (Block 1)**		**Model 2 (Block 1 and 2)**		**Model 3 (Block 1 and 3)**	
	**R**^**2**^	**β**	**R**^**2**^	**β**	**R**^**2**^	**β**
**Block 1:Perceptions**	.49		.49		.59	
Consequences		–.55^***^		–.55^***^		–.50^***^
Timeline		–.16		–.14		–.08
Personal control		–.09		–.08		–.06
Treatment control		.16^*^		.16^*^		.20^**^
Identity		–.09		.11		–.06
Illness concern		.01		.02		.06
Comprehensibility		.07		.04		. 07
Emotional response		–.15		–.14		-.17
**Block 2: Demographic characteristics**						
Age				–.01		
Gender				–.10		
**Block 3: Clinical characteristics**						
MRC dyspnoea						–.35^**^
FEV%pred						–.11
Comorbidity						.05
**F change**		11.25^***^		7.69		6.47^***^

R^2^ is an adjusted R^2^. β is a standardised β.

*P ≤ 0.05; **P ≤ 0.01; ***p < 0.001.

FEV%pred = forced expiratory volume percentage from predicted, MRC dyspnoea = Medical Research Council dyspnoea scale.

As shown in Table 6 (model 1, 2 and 3), *treatment control* (β = .19) and *emotional response* (β = -.33 - –.40) were associated with the CRQ-SAS’s emotional domain. *Treatment control, emotional response* and dyspnoea explained 35% of the variation in the CRQ-SAS’s emotional domain. These results indicate that COPD patients with better treatment control and a weaker emotional response to their disease have better HRQoL as measured by the CRQ-SAS’s emotional domain (the mean of the emotional function and mastery domains).

**Table 6:** Regression models between separate illness perception items and dependent variable health-related quality of life (CRQ emotional) N = 87

	**Model 1 (Block 1)**		**Model 2 (Block 1 and 2)**			**Model 3 (Block 1 and 3)**
	**R**^**2**^	**β**	**R**^**2**^	**β**	**R**^**2**^	**β**
**Block 1:Perceptions**	.32		.32		.35	
Consequences		–.17		-.17		–.14
Timeline		.01		.03		.05
Personal control		.03		.05		.05
Treatment control		.17		.17		.19^*^
Identity		–.08		-.10		
Illness concern		–.03		-.01		
Comprehensibility		–.53		-.09		
Emotional response		–.40^**^		–.39^**^		–.33^*^
**Block 2: Demographic characteristics**						
Age				–.02		
Gender				–.14		
**Block 3: Clinical characteristics**						
MRC dyspnoea						–.23^*^
FEV%pred						–.10
Comorbidity						.05
**F change model**		6.01^***^		1.08		1.74

R^2^ is an adjusted R^2^. β is a standardised β.

* P ≤ 0.05; ** P ≤ 0.01; *** p < 0.001.

FEV%pred = forced expiratory volume percentage from predicted, MRC dyspnoea = Medical Research Council dyspnoea scale.

## Discussion

This study shows that specific dimensions of illness perceptions are associated with HRQoL in COPD patients with mild to severe COPD (GOLD I-III) who receive medical support from a primary care physician and a practice nurse in primary care. COPD patients have better HRQoL when they experience fewer symptoms attributed to COPD (identity), experience less impact in daily life (consequences), experience fewer emotional consequences (emotional response), have stronger beliefs about control of their treatment and have a greater understanding of the disease (comprehensibility). When corrected for dyspnoea, airflow limitation and comorbidity, *identity, comprehensibility* and dyspnoea explained 56% of the variation in HRQoL (CCQ). *Consequences, treatment control* and dyspnoea explained 59% of the variation in HRQoL (the CRQ-SAS’s physical domain), and *emotional response* and dyspnoea explained 35% of the variance in HRQoL (the CRQ-SAS’s emotional domain).

The findings of our study are in line with the results of other studies regarding illness perceptions in COPD patients. Scharloo [28] and colleagues have concluded that outpatient COPD patients who have a strong illness identity and strong beliefs regarding the consequences of their illness have worse general functioning and HRQoL. In another study by Scharloo [16] of outpatient COPD patients, decreased symptoms, more positive beliefs about the effects and outcomes of treatment and less strong emotional reactions were associated with higher HRQoL. Our data support these findings. Our findings are also in line with findings regarding illness perceptions in COPD patients undergoing rehabilitation; more positive and adaptive attitudes about treatment are related to better outcomes and general functioning [17,29,30].

Although some studies show that coping with illness mediates the relationship between illness perceptions and the overall outcome of the illness [13], our previous research [9] and research by Heijmans et al. [31,32] has revealed that HRQoL is more influenced by illness perceptions than by coping strategies.

Our study lends support to the Common Sense Model (CSM), [12] which suggests that people hold views and beliefs about their illness that are associated with their HRQoL. These illness perceptions are the key elements for understanding how people manage threats to their health and experience their HRQoL [12].

To appreciate the findings of this study, some aspects require further consideration. The current study has some limitations. First, because of the cross-sectional nature of this study, the associations between the dimensions of illness perceptions and HRQoL should not be understood as implying a causal relationship. In light of these findings, it is important to address the possibility of some conceptual overlap between the specific dimensions of illness perceptions and HRQoL. There were some significant correlations between the illness perception dimensions and the HRQoL measures, but they were too low to determine collinearity. Longitudinal data will enable us to explain the relationship between illness perceptions and HRQoL in more detail. Second, the βs in the regression models were small, indicating small clinical changes per unit change. Third, the sample size did not allow for subgroup analyses per GOLD grade. Therefore, we could not describe the associations in the different stages of COPD. Furthermore all measures were questionnaires. It could be questioned whether a questionnaire is the best measure of illness perceptions because the development of perceptions is partially an unconscious process. Qualitative interviews might be preferable to questionnaires. However, the aim of the study was to quantify the relationship between illness perceptions and HRQoL, for which regression analysis is the preferred method. Furthermore, patient-reported outcome measures of illness perceptions (B-IPQ) [19,33] and HRQoL [34] have been shown to be valid and reliable.

The strength of the present study is its generalisability. In our study population, 20% of the patients had mild COPD (GOLD grade I), and almost 70% of the patients had moderate COPD (GOLD grade II). This sample is in line with the distribution of COPD patients in primary care [4]. Moreover, we did not exclude patients with comorbidities. Therefore, our study population is representative of the primary care population.

## Conclusions

This study highlights the importance of patients’ beliefs about their illness and symptoms in relation to HRQoL. The results of this study indicate that the HRQoL of COPD patients is associated with illness perceptions together with the severity of dyspnoea as experienced by patients. More objective measures, such as airflow limitation measures or comorbidities, do not add to the explanation of HRQoL*.*

A major goal of COPD treatment is to improve HRQoL [1], and this study contributes to the existing knowledge concerning the associations between illness perceptions and HRQoL. Despite their importance, patients’ beliefs and views of their symptoms and illness are rarely discussed in consultations [14]. The results of this study confirm the existing knowledge and provide starting points for the development of interventions focusing on illness perceptions both to support COPD patients in their disease management and to improve HRQoL. Because evidence suggests that the degree of physician-patient concordance regarding perceptions of symptoms is poor [35,36] and that addressing illness perceptions is more important than interpersonal skills in relation to patient adherence [37], the starting point should be to explore these illness perceptions (e.g., with questionnaires) [38]. The second step should be to discuss these perceptions and, if necessary, to correct them at an early stage. Realistic positive beliefs should be encouraged, and negative beliefs should be prevented or challenged [18,39,40]. This approach may result in better HRQoL in COPD patients. Because interventions focusing on illness perceptions have only recently been described in patients with other chronic diseases [41-43], it is important to develop and test an illness perception intervention for COPD patients in primary care settings.

## Competing interests

The authors declare that they have no competing interests.

## Authors’ contributions

SW contributed to the study concept and design, data collection, data analysis, and writing of the manuscript and takes full responsibility for the integrity of the data and the accuracy of the data analysis. JL contributed to the study concept and design, providing input on the data analysis, reviewing, and final editing of the manuscript. MH provided input on the data analysis, reviewing and editing of the manuscript. MS contributed to the study concept and design, providing input on the data analysis, reviewing, and final editing of the manuscript. All authors read and approved the final manuscript.

## Authors’ information

SW is a researcher and a nurse in the Heart & Lungs Division at the University Medical Center Utrecht. As a clinical nurse specialist in lung diseases, she has extensive experience in the care and guidance of COPD patients. She is currently working on her PhD research project. The aim of this project is to develop and test a feasible illness perception intervention for COPD patients to be applied by practice nurses in a primary care setting. This nursing intervention is being evaluated in a cluster randomised trial that began in the spring of 2013. In her previous projects, she has gained experience with developing interventions to improve self-management and decrease functional decline.

JWL is a pulmonologist and head of the Department of Respiratory Medicine of the University Medical Center Utrecht. Amongst other clinical and preclinical studies directed at COPD, including the TiPharma projects on COPD and the COPACETIC study (a KP7 project), he has been involved in studies of patient adherence, illness perceptions and medication adherence in patients with pulmonary diseases.

MH is a senior researcher at the Netherlands Institute for Health Services Research (NIVEL) and at NPCG: National Panel of the Chronically Ill and Disabled. Her research topics are self-management of the chronically ill, participation and quality of life.

MS is a researcher, a nurse and an appointed professor in Nursing Science at the University Medical Center Utrecht. She is also a Professor of Care for Older People at the University of Applied Sciences in Utrecht. Her research focuses on the daily functioning of older people with multiple morbidities.

## Pre-publication history

The pre-publication history for this paper can be accessed here:

http://www.biomedcentral.com/1471-2296/15/140/prepub
